# Preliminary study for dose evaluation depending on dose range with optically stimulated luminescence dosimeter considering individual dosimeter sensitivity

**DOI:** 10.1371/journal.pone.0266110

**Published:** 2022-03-29

**Authors:** Su Chul Han

**Affiliations:** Department of Radiation Oncology, Yonsei University College of Medicine, Seoul, Korea; Stanford University School of Medicine, UNITED STATES

## Abstract

The purpose of this study was to investigate dose evaluation depending on dose range using optically stimulated luminescence dosimeter (OSLD) and evaluate the possibility of high dose evaluation. This study investigated a commercial OSLD and used a Co-60 gamma irradiator for irradiation. The OSLDs (N = 26) were sampled in total OSLDs (N = 46) depending on the radiation sensitivity for this study. After irradiating doses from 0.5 to 40 Gy at fixed intervals in a standard environment, the dose response of a reference OSLD (N = 5) was determined through the reading process at each dose. The dose-response curves obtained from the reference OSLD were fitted according to the dose. In the dose range below 3 Gy, a linear function was used to determine the relationship between dose and the OSLD response. Quadratic and cubic functions were applied for dose ranges of up to 15 Gy and 40 Gy, respectively. Test OSLDs (N = 21) were evaluated at various doses (2.5 to 30 Gy) using different fitting functions, according to dose ranges. When doses from 0.5 Gy to 3.0 Gy were curve-fitted to the linear function, the relationship was y = 70278.0*x* − 3125.3 (r^2^ = 0.999). When doses of up to 15 Gy were curve-fitted to the quadratic function, the relationship was y = 628.6*x*^2^ + 70444.6*x* − 6142.3 (r^2^ = 0.999). Furthermore, when doses of up to 40 Gy were curve-fitted to the cubic function, the relation was y = −15.5*x*^3^ + 527.3*x*^2^ + 75059.6*x* − 16260.3 (r^2^ = 0.998). Test OSLDs were evaluated for various dose ranges based on the above equation. It was confirmed that the average difference was 0.86 ± 0.27%, and it was evaluated that the largest difference occurred at 30 Gy (2.24 ± 0.24%). In this study, we prove that measurements using the OSLD at various dose ranges, including high doses, will be possible through the application of an in-house software program and a correction process.

## Introduction

An optically stimulated luminescence dosimeter (OSLD) has a shorter processing time for dose measurement than the thermoluminescent dosimeter (TLD) and it has the ability to take repeated readings [1]. Furthermore, the OSLD is a useful tool for measuring skin dose [2], and it has small chips that make it easier to use than the radio-photoluminescence glass rod dosimeter (RPLGD) [3]. Studies have been conducted to assess patient dose among various OSLD energy ranges from diagnostic to therapeutic radiation. For example, Bao et al. [4] used an OSLD to verify the patient skin dose in total skin electron irradiation (TSEI). Wake et al. [5] also used an OSLD to measure the skin dose of patients who received post-mastectomy radiation therapy. Furthermore, many studies have been conducted to evaluate the skin dose using OSLDs in low-energy diagnostic radiation and high-energy radiation therapy [2, 6–8]. To evaluate patient skin dose using OSLDs in various energy domains, studies evaluating the dose characteristics of OSLDs must be performed first, and many researchers have conducted these studies. For example, Jursinic et al [1] studied the reproducibility of OSLDs, including the homogeneity and linearity of arrangements for photon and electron beams and Ir-192 sources. Kerns et al. [9] evaluated the linearity and reproducibility of proton beams and evaluated the incident angle dependence by simulating OSLDs. Kim et al. [10], Hoshida et al. [11] and Ito et al. [12] evaluated the dose response of OSLD in megavoltage. Al-Senan et al. [13] investigated the dosimetric characteristics of diagnostic radiography.

However, while OSLDs have many advantages, they also suffer from quite a few disadvantages in taking dose measurements. For example, their radiation sensitivity is affected by accumulated doses. Jursinic et al. [14] evaluated changes in radiation sensitivity by irradiating accumulated doses of up to 60 Gy and certified the sensitivity using irradiation of OSLDs with greater than 1 kGy; similarly, Reft et al. [15] also irradiated accumulated doses of up to 60 Gy. Currently, it is not recommended to utilize OSLDs in clinical settings above the range of 15 Gy. For instance, the AAPM-TG-191 recently recommended using up to 10 Gy in the case of reuse [16].

However, the development of radiotherapy units with high technologies has resulted in changes in the trend of treatment technologies that irradiate tumors with high doses such as SRS(Stereotactic Radiosurgery) and SBRT(Stereotactic Body Radiation Therapy), and many methods have been suggested for delivering quality assurance before radiotherapy.

This study obtained the dose-response curves of OSLDs from 0.5 Gy to 40 Gy, which were fitted according to the dose range to evaluate the dose in radiotherapy. Furthermore, an in-house software was developed to evaluate the dose according to the dose range using OSLDs.

## Material and methods

### Dosimeters and measuring system

**Fig 1:**
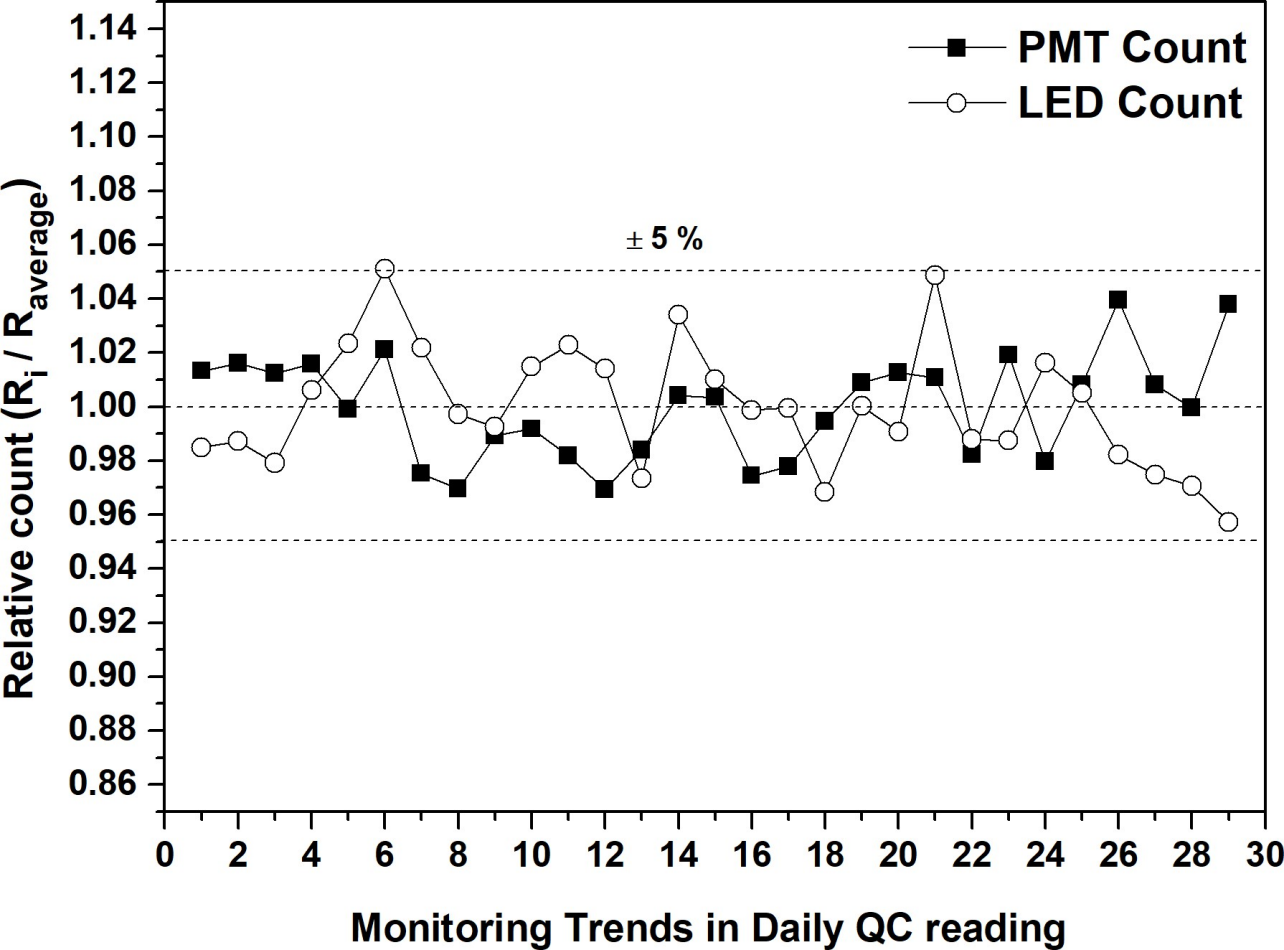
Monitoring trends of reader stability during our study, where *R*_*average*_ is the average signal of the reading system during monitoring, and *R*_*i*_ denotes the ith signal.

This study investigated a commercial OSLD (Nano Dot, Landauer Inc., Glenwood, USA) and used a Co-60 gamma irradiator (Theraton 780, AECL, and Kanda, Canada) for irradiation. To read the irradiated dosimeter, the in Light MicroStar reader (Landauer Inc., Glenwood, USA) of the continuous wave optically simulated luminescence (CW-OSL) mode was used. The uniqueness of the reading system was checked to verify the stabilization of the reader. The checking items included dark current (DRK), radiation measurement of the C-14 source embedded in the reader (CAL), and the beam strength stability evaluation (LED). The results showed that the DRK value was maintained below 10 during the experiment, and the other two items were maintained within 5% (Fig 1). For the optically annealed system of the dosimeters, a 24 W fluorescent lamp with a wavelength of 280–780 nm, manufactured by Hanil Nuclear Co., Ltd, was used. The dosimeter reading and optical annealing processes were performed using the results obtained from a previous study [17].

### Determination of dose-response curve according to dose range

To verify the batch sensitivity and reproducibility of the dosimeters used in this study, the batch sensitivity of each dosimeter was conducted with 46 dosimeters as one batch. In the batch, the process (irradiation-reading-optical annealing) was performed four times under the following conditions: a source surface distance of 80 cm, field size of 10 cm × 10 cm, and depth of 0.5 cm. The response signal of the OSLD was defined in this study. The batch sensitivity between the dosimeters in the batch was calculated using Eq (1):

$$Batch\;sensitivity=\frac{C_{n}}{\bar{C_{batch}}}=\frac{C_{n}}{\frac{1}{N}\sum_{n=1}^{N}C_{n}}=\frac{\frac{1}{R}\sum_{r=1}^{R}C_{n,r}}{\frac{1}{N}\sum_{n=1}^{N}\sum_{r=1}^{R}C_{n,r}},$$ (1)

where *c*_*n*_ is the count value of the nth dosimeter when 1 Gy is irradiated, and $\bar{c_{batch}}$ is the average value of the batch (average of the values acquired when the dosimeters in one batch were irradiated at 1 Gy). R indicates the total number of reading repetitions during the on-process (R = 3), and N indicates the total number of dosimeters evaluated in this study. To correct the sensitivity between dosimeters, the element correction factor (ECF) can be used as described in another study [18]. Before we irradiated the dose, we evaluated it according to TRS −398 with a farmer-type chamber (PTW, TN30013) and an electrometer in a water phantom. The farmer-type chamber was calibrated using the SSDL of Korea.

The OSLD generally has a linearity of up to 3 Gy or 2.5 Gy and has a supra linearity at higher doses. Furthermore, the manufacturer of the OSLD provides a nonlinear mode for the correction of supra linearity, and the dose range for this is 15 Gy [19]. Considering these, we fitted the dose-response curve using the linear, quadratic, cubic functions until the doses of 3 Gy, 15 Gy, and above 15 Gy, respectively. The 60-Co gamma ray was used to determine the dose-response curve as follows: We selected reference dosimeters in one batch whose sensitivity was close to the average value ($\bar{c_{batch}}$). The relationship between a known dose (*x*) and the response signal (Y, count) was evaluated using reference dosimeters with a low reproducibility variation coefficient.

After irradiating doses from 0.5 to 40 Gy at fixed intervals in a standard environment, the count value of the OSLD was determined through the reading process at each dose. Moreover, the relationship for the dose-response curve was determined while changing the curve fitting function according to the dose range in Eq (2).

$$Y(Count)=\left\{ \begin{array}{ll} \;a_{0}x+b_{0} & \left(0.5<x\leq 3.0\right)\\ \;a_{1}x^{2}+b_{1}x+c_{1} & \left(3.0<x\leq 15.0\right)\\ a_{2}x^{3}+b_{2}x^{2}+c_{2}x+d_{2} & \left(15.0<x<40.0\right) \end{array},\right.$$ (2)

where *x* is the known dose determined in the water phantom, and Y is the response signal of the OSLD. *a*_0,1,2,_ *b*_0,1,2,_, *c*_1,2,_ and *d*_2_, are constants determined by the fitting function.

### Dose evaluation with dose-response curve according to dose range

We tested the dosimeters for doses higher than 15 Gy (20 and 30 Gy, N = 6) and evaluated them under a 15 Gy dose (2.5, 3, 5, 13, 15 Gy, N = 15). The total number of dosimeters (N = 21) was used in sampling OSLDs for dose evaluation using dose-response curves according to the dose range.

The raw count (*C*_*raw*_) of OSLDs measured in test doses was adjusted as the corrected count (*C*_*corr*_) using various correction factors (sensitivity, reading system, and exposure), according to Eq 3. Because the test dosimeters were irradiated in a standard environment with specific parameters such as the exposure condition of the reference dosimeter, the exposure condition correction factor (*E*_*f*_) was defined as 1.00. The reading system correction factor (*R*_*f*_) is the uncertainty generated in the OSLD reading process and because all reading processes were performed as similarly as possible to our previous study [7], the reading system correction factor was determined as 1.00. The sensitivity correction factor (*S*_*f*_) refers to the sensitivity of the reference dosimeters to determine the dose-response curve and the ratio of the test dosimeters for dose evaluation in Eq (4).


$$C_{corr}=C_{raw}\times S_{f}\times R_{f}\times E_{f}$$ (3)

$$Sensitivity\;correction\;factor\;(S_{f})=\frac{Batch\;{Sensitivity}_{ref.\;dosimeters}}{{Batch\;Sensitivity}_{test\;dosimeters}}=\frac{S_{ref.}}{S_{test}}$$ (4)

**Fig 2:**
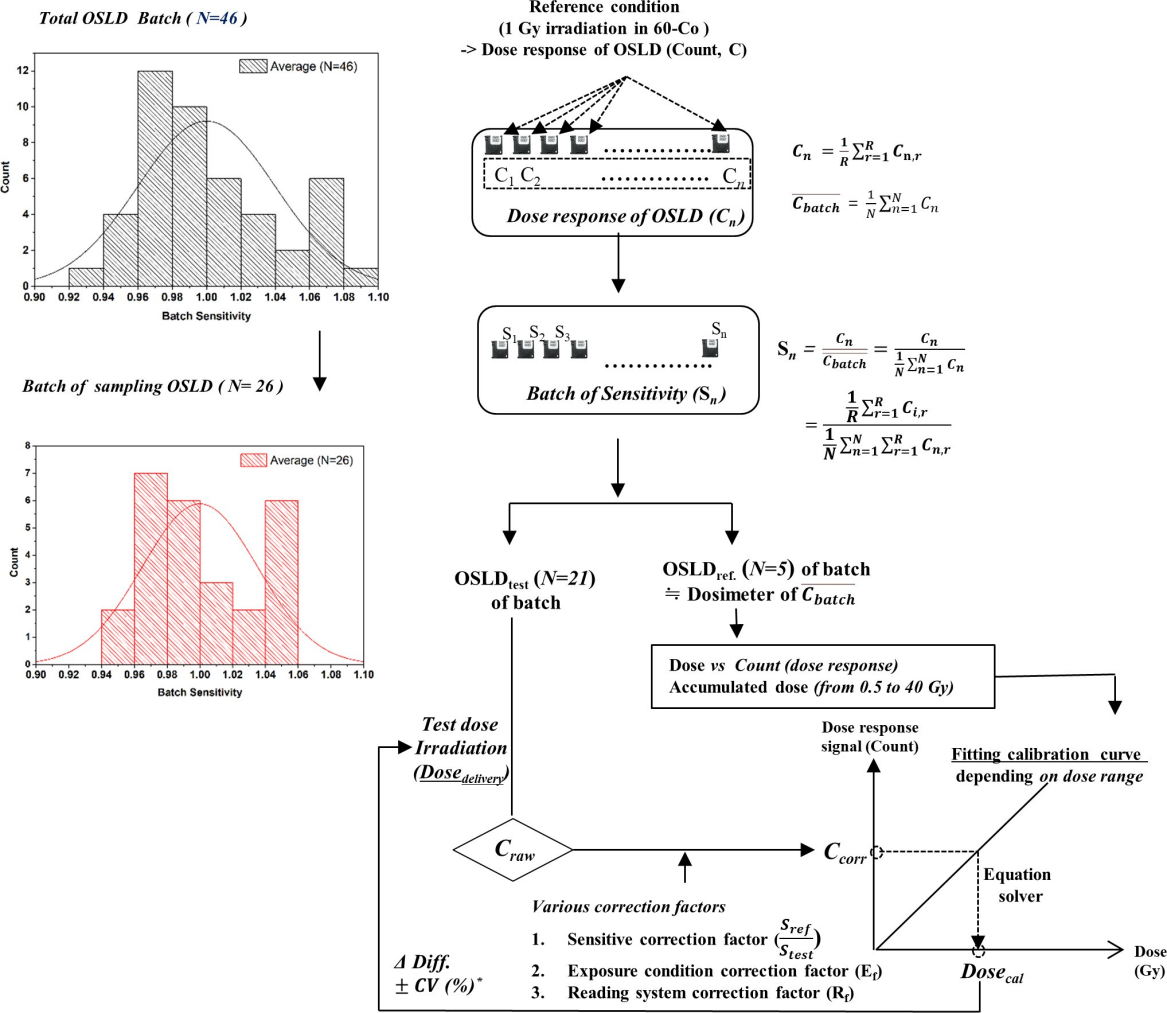
Overall concept for dose evaluation with OSLD depending on dose range.

Finally, the solution (unknown dose, *x*) can be obtained by Eq (2), in which *C*_*corr*_ was inputted instead of Y. Fig 2 shows the overall concept of the dose evaluation performed using OSLD in this study.

An in-house software program based on the dose-response curve of the OSLD was developed using LabVIEW. The program was designed to include the following three components:

First, to obtain data for determining the dose-response curve, the program was designed to receive the known dose and the acquired count values as input and acquire a fitting function according to the dose range for the relationship between the dose and the dose response (count). Second, it was designed to correct the measured count acquired from the test dosimeters irradiated with a dose. The sensitivity correction factor, exposure condition correction factor (the energy, angular correction factor), and the reading system correction factor (the fading and depletion correction factors) were used to correct the sensitivity of the dosimeters used for testing and determining the dose-response curve. Third, it was designed to inversely calculate the dose by applying the count value corrected by the various correction factors to the fitting function of the dose-response curve determined by the dose range. The fitting function was selected based on the target dose and the target. Furthermore, the target and calculated doses were compared, and the percentage difference was calculated using Eq (5):

$$Dose\;Diff.(\Delta_{dose},\%)=\frac{D_{target}-D_{eval.}}{D_{target}}\times 100,$$ (5)

where *D*_*target*_ (known dose) is the dose irradiated onto the dosimeter, and *D*_*eval*._ is obtained from the count value measured from the OSLD using the in-house program.

Δ_*dose* (*n*)_±*CV*_*n*_ was defined considering the coefficient of variation (CV) of reproducibility for 1 Gy of test dosimeter in our study, where *n* is the number of test dosimeters in each dose. To estimate the average dose difference in each dose, we used their known values and calculated the average value of the dose difference; μ means SD, considering the reproducibility of each OSLD (Eq (6)).


$$Average\;dose\;diff.in\;each\;(\bar{Average\pm \mu })=\frac{1}{N}\sum_{n=1}^{N}\Delta_{dose(n)}\pm {CV}_{n}(N=3)=\frac{1}{3}(|\Delta_{dose(1)}|+|\Delta_{dose(2)}|+|\Delta_{dose(3)}|)\pm \frac{1}{3}\sqrt{{CV}_{1}^{2}+{CV}_{2}^{2}+{CV}_{3}^{2}}$$ (6)

## Results

### Evaluation of batch sensitivity and reproducibility of OSLD

**Fig 3:**
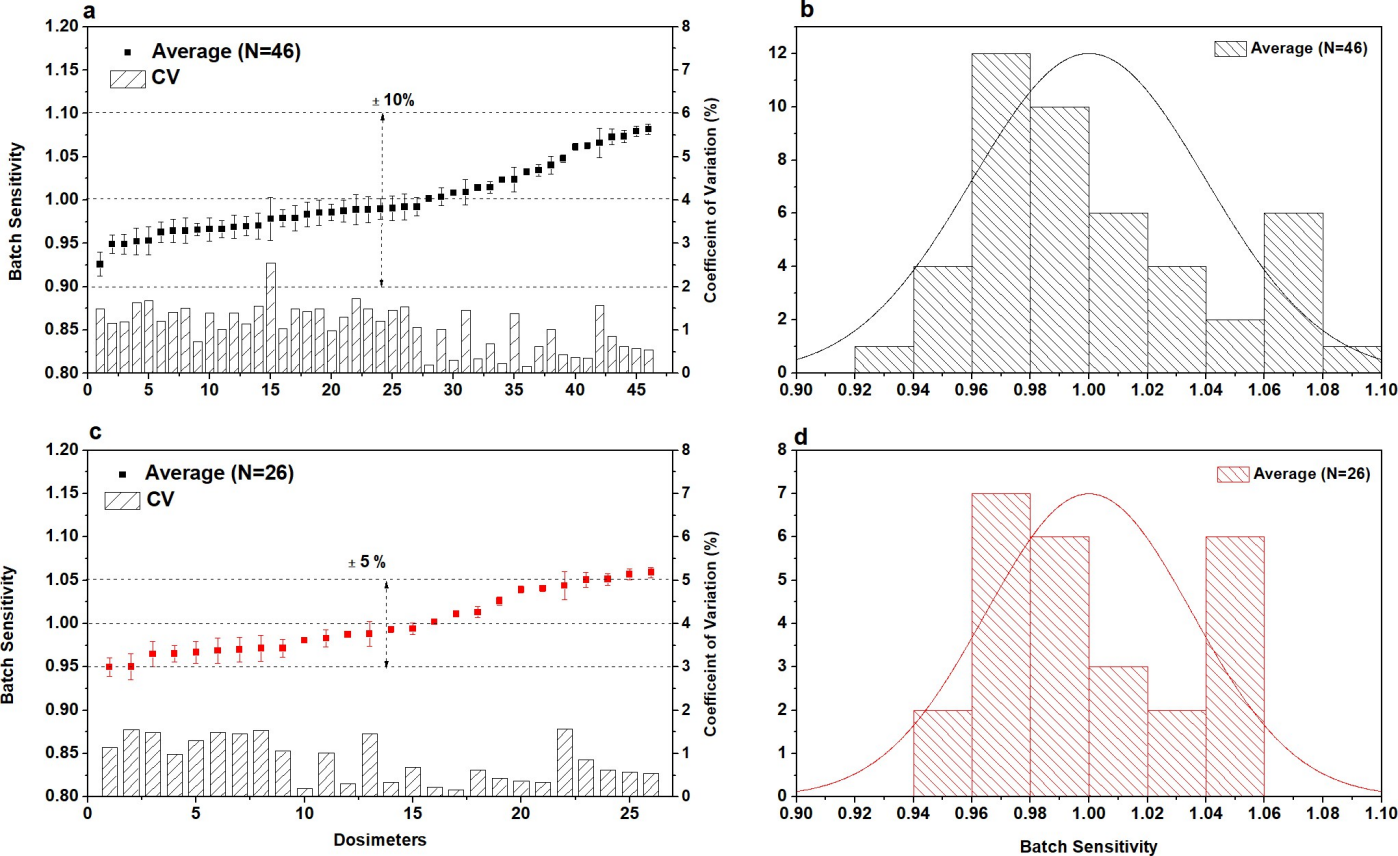
Comparison of batch sensitivity and coefficient of variation (CV) for reproducibility in this study. (a) Total OSLDs in one batch (N = 46); (b) Histogram of Total OSLDs (frequency); (c) Sampling OSLDs in one batch (N = 26); and (d) Histogram of Sampling OSLDs in one batch (frequency).

The 46 dosimeters used in this study were considered as one batch (N = 46), and Fig 3 shows their batch sensitivities, which range from 0.93 to 1.09 (within ±10%). To carry out this study, the dosimeters of the batch, whose sensitivity was less than approximately ±5%, were sampled (N = 26), and the reference dosimeters (N = 5) were selected to evaluate the dose response of the OSLD.

### Determination of dose-response curve according to dose range and evaluation of adjacent doses

The reference dosimeters (N = 5), which were selected considering batch sensitivity (within ± 2%) and reproducibility (within 0.5%), were irradiated from 0.5 to 40 Gy. The reference dosimeters were read in each dose (15 points for dose) after they were stabilized. To irradiate each dose in the reference dosimeter, they were cumulatively irradiated in intervals of 0.5 Gy up to 3 Gy, and were then irradiated at additional doses of 1, 2, 3, 5, and 10 Gy (Table 1). The dose-response curve was determined from the relationship between the dose and the count (the average and standard deviation) measured using the reference dosimeters for each dose. The difference between the dosimeters at each dose was less than 1.5%, showing the same response to radiation (Table 1). Furthermore, after the interval dose was added to each dose, the count values were compared. It was found that the count values accumulated at constant intervals for the added dose of 0.5 Gy, excluding the dose ranges of 2.5 Gy to ~3 Gy. When the same dose was applied, the accumulated count value increased in the dose ranges of 3–15 Gy. Finally, for the total doses above 15 Gy, the accumulated count value decreased even though the same dose (5 Gy) was used.

**Table 1:** Relationship between dose and count value used to determine the dose-response curve (“Average” and “SD” indicate the average and standard deviation of reference dosimeters (N = 5), respectively, and “Interval dose” means a dose added between the doses; CV means variation of measured count value in each dose with the reference OSLD.

Accumulated total dose (Gy)	Average ± S.D (Count, N = 5)	Interval dose (Gy)	Interval count	CV (%)
0.5	33369 ± 205	0.5	33231	0.61
1.0	66734 ± 800	0.5	32879	1.19
1.5	101375 ± 1284	0.5	34346	1.26
2.0	136583 ± 1345	0.5	35509	0.98
2.5	171913 ± 1767	0.5	35009	1.02
3.0	209193 ± 1880	0.5	39669	0.89
4.0	281685 ± 3839	1.0	69229	1.37
5.0	357830 ± 3199	1.0	76957	0.89
7.0	518281 ± 6063	2.0	162398	1.16
10.0	769592 ± 9390	3.0	241988	1.23
15.0	1188948 ± 11727	5.0	429632	0.98
20.0	1589653 ± 13569	5.0	397285	0.85
25.0	1952959 ± 17856	5.0	367160	0.91
30.0	2264157 ± 20341	5.0	305798	0.89
40.0	2841354 ± 15487	10.0	575663	0.83

**Fig 4:**
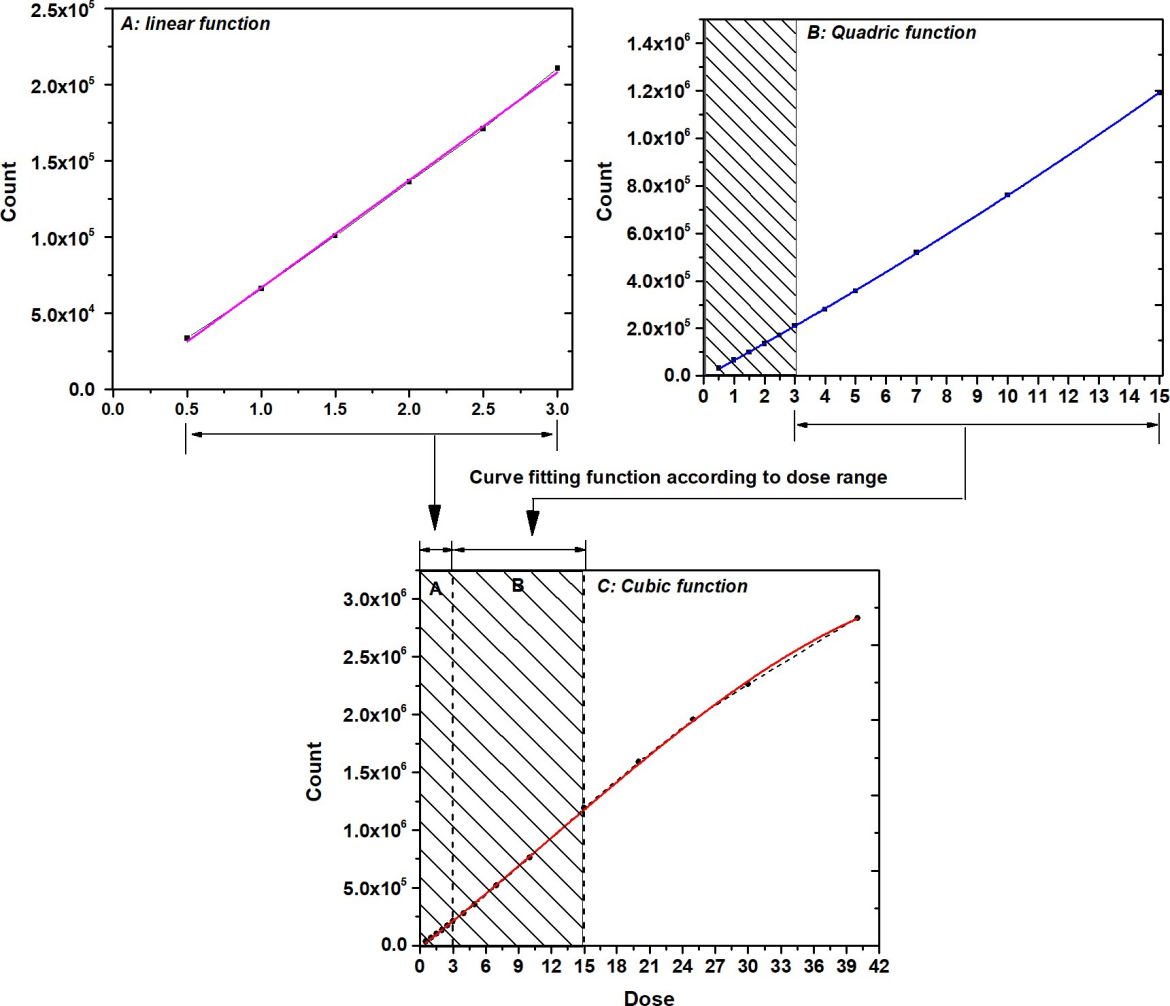
Curve fitting functions of the dose-response curve for dose evaluation according to the dose range of the OSLD. A: Linear function, B: Quadratic function, C: Cubic function.

Different fitting functions were applied according to the dose ranges for the relationship between the delivered dose and the dose response of OSLD (count). When doses from 0.5 Gy to 3.0 Gy were curve-fitted to the linear function, the relation was y = 70278.0*x* − 3125.3 (r^2^ = 0.999). When doses up to 15 Gy were curve-fitted to the quadratic function, the relationship was y = 628.6*x*^2^ + 7044.6*x* − 6142.3(r^2^ = 0.999). Furthermore, when doses of up to 40 Gy were curve-fitted to the cubic function, the relation was y = −15.5*x*^3^ + 527.3*x*^2^ + 75059.6*x* − 16260.3 (r^2^ = 0.998). Finally, the compound fitting function can be calculated by extracting the curve that is fitted for each dose range (Fig 4).

### Dose evaluation using the in-house program

**Fig 5:**
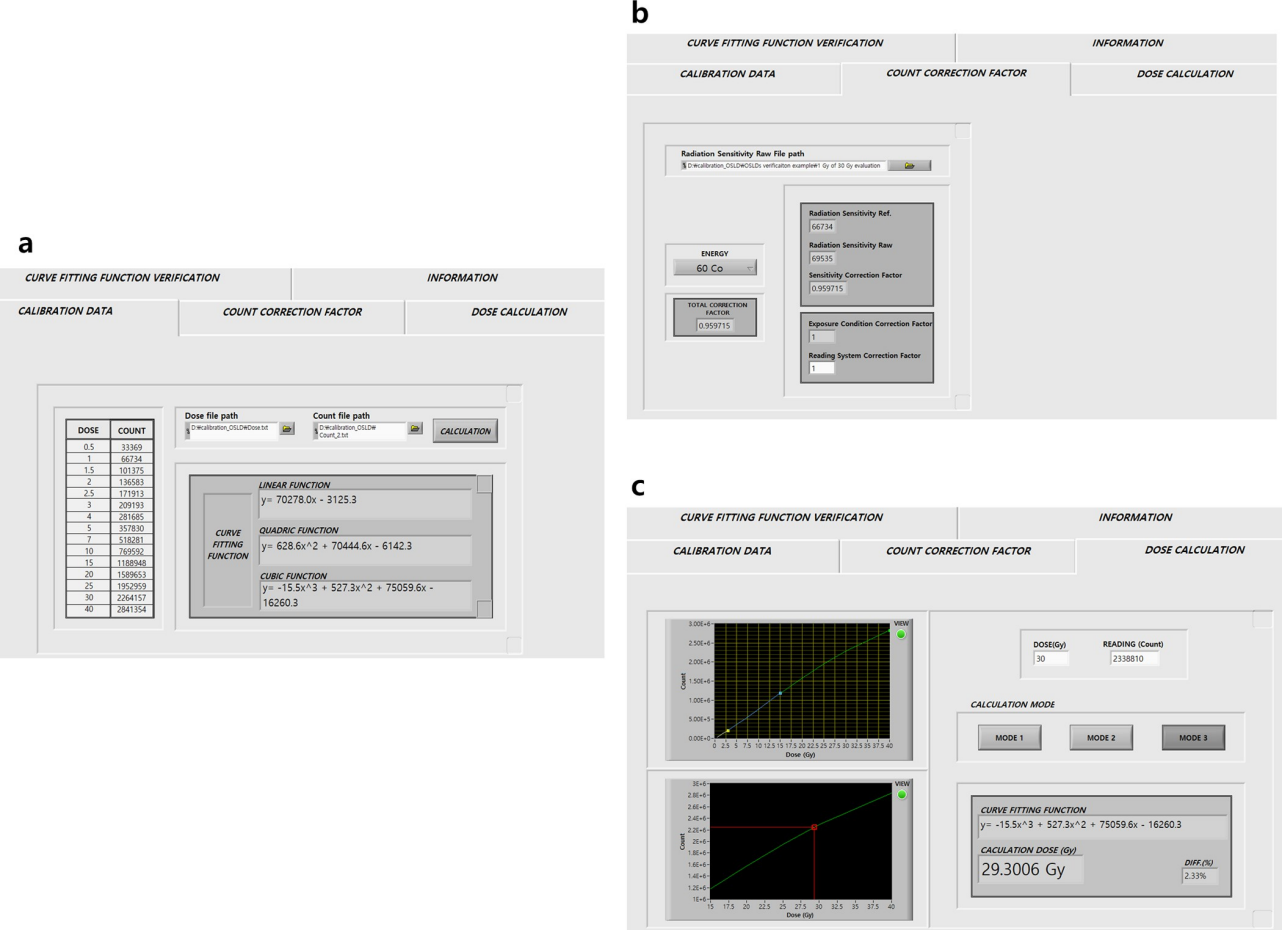
GUI of the OSLD dose evaluation program (example for 30 Gy). (a) Information entered to determine the dose-response curve and fitting function according to the dose range, (b) count corrected using the correction factor from the raw count, and (c) dose evaluation result for the corrected count using the program.

An example of the dose evaluation process for the target dose (30 Gy) delivered by Co-60 gamma rays using the developed program is shown in Fig 5. The known dose and count values (dose response of OSLDs) were inputted as files to determine the dose-response curve, and the relation was obtained as output by applying different fitting functions depending on the dose range. Then, the Co-60 gamma ray was selected for the delivered energy (Fig 5A), and the sensitivity correction factor (the value was 0.959 in the used test OSLD) was calculated by inputting the radiation sensitivity of the test dosimeter. The values of the exposure condition factor and the reading system correction factor were entered as 1.00 for this study (Fig 5B). Finally, 30 Gy was entered in the predicted dose (target dose) and the measured value (*C*_*raw*_) was entered when irradiated with 30 Gy. In Fig 5C, the y-axis represents the corrected value (*C*_*corr*_) using the correction factors, and the x-axis represents the calculated dose (*D*_*Cal*._) obtained using a fitting function. Consequently, the value of the difference between the predicted dose (30 Gy) and the calculated dose (29.30 Gy) was 2.33% at 30 Gy.

Table 2 shows the doses in a specific range (2.5 to ~30 Gy) for 21 test OSLDs and shows the calculated results by applying different fitting functions according to the dose. After irradiating each target dose to the test OSLDs (N = 3) under standard exposure conditions, the measured count from each OSLD was inputted into the developed program to calculate the dose. Furthermore, the difference was analyzed by comparing the target and evaluated doses using the program, considering the uncertainty of the variation in the reproducibility of the OSLD. The variation range of the reproducibility for the test OSLD ranged from 0.55% to 1.82%, and the average was 1.09±0.38%.

**Table 2:** Difference between the target and evaluated doses in various doses (from 2.5 Gy to 30 Gy) determined using the in-house software program (“Average” and “SD” indicate the average and standard deviation of the test dosimeters (N = 3) in each dose; CV means variation of the reproducibility for each test dosimeter).

*Dose*_*target*_ (Gy)	Test dosimeters (S_f_) $\left(\frac{S_{ref}}{S_{test}}\right)$	*Dose*_*eval*._ (Gy)	Fitting function	Δ_*dose*_ ± CV (%)	Average Dose Diff.(%) ($\bar{Average\pm \mu }$)
2.5	1.048	2.496	Linear	0.17 ± 1.14	0.64 ± 0.76
	1.027	2.539		−1.56 ± 1.49	
	1.029	2.495		0.21 ± 1.30	
3.0	1.012	3.015	Linear	−0.48 ± 1.02	0.59 ± 0.59
	1.024	3.018		−0.59 ± 1.05	
	1.031	3.021		−0.70 ± 0.99	
5.0	0.950	4.990	Quadric	0.20 ± 1.07	0.71 ± 0.74
	0.958	5.006		−0.12 ± 0.64	
	0.958	5.091		−1.82 ± 1.82	
13.0	1.024	12.878	Quadric	0.94 ± 1.53	0.63 ± 0.88
	1.031	12.958		0.33 ± 1.49	
	1.047	12.920		0.62 ± 1.56	
15.0	1.026	15.026	Quadric	−0.17 ± 1.46	0.33 ± 0.93
	1.001	15.050		−0.33 ± 0.68	
	1.007	14.926		0.62 ± 1.45	
20.0	0.949	20.174	Cubic	−0.87 ± 0.76	0.90 ± 0.50
	0.943	20.146		−0.73 ± 0.94	
	0.944	20.220		−1.10 ± 0.88	
30.0	0.971	30.265	Cubic	−0.88 ± 0.55	2.24 ± 0.34
	0.983	31.050		−3.50 ± 0.56	
	0.959	29.031		2.33 ± 0.61	

The smallest difference between the target dose and the calculated dose was in the 15 Gy batch (0.33 ± 0.93%), and the largest difference was found in the 30 Gy batch (2.24 ± 0.34%). The average difference (Average±μ) was 0.86 ± 0.27%, regardless of the target dose.

## Discussion and conclusion

The OSLD was evaluated using high doses and an in-house software program developed for high-dose evaluation. Generally, OSLDs have a linearity of up to 3 Gy or 2.5 Gy and possess phase linearity at higher doses. Considering this, manufacturers support the non-linear mode for doses higher than 3 Gy. However, we conducted this study by considering the possibility of measuring doses higher than this. We presumed that it would be sufficiently possible if the dosimeter showed a similar tendency at doses higher than a specific value.

Luminance dosimeters such as TLD and OSLD have batch sensitivities that show different responses, even if they are manufactured in the same way. Furthermore, if the batch sensitivity is not checked before using the dosimeter, different results may be obtained even if the same dose is irradiated. Therefore, the TRS-457 protocol recommends correcting each dosimeter of TLD when doses are tested in diagnostic radiology [20]. In contrast, manufacturers of OSLDs provide screened dosimeters to users, but the difference in radiation sensitivity between the screened dosimeters is less than 5% [16].

The accuracy of measurements can be improved by verifying in advance the sensitivity for the OSLDs introduced in each organization. In this study, the sensitivity data for dosimeters were acquired by verifying the sensitivity of dosimeters that had not been previously irradiated, and the entire study was conducted based on this result. Furthermore, the dose–response curve was determined by selecting reference dosimeters in a single batch. When using dosimeters other than those among the reference dosimeters, the dose was calculated by applying a correction factor for the sensitivity of the dosimeters. Studies by other researchers have demonstrated changes in dosimeter sensitivity caused by accumulated and high doses [14, 21, 22]. OSLDs that received high doses or cumulative doses have limitations in reuse due to changes in radiation sensitivity. Consequently, reuse is not recommended if a specific minimum dose or more is irradiated. Hence, radiation sensitivity is important in dosimetry with OSLDs.

Current radiation therapy uses higher doses than before. An OSLD may not be used to measure the point dose inside a phantom. However, this is possible if reference dosimeters are selected, and the dose-response curve of a high dose under standard conditions can be designed using the selected reference dosimeters. Furthermore, in this study, we designed it to use the most appropriate dose-response curve according to the dose range, instead of using the same dose-response curve up to a high dose for all dose ranges.

We improved the accuracy of the dose evaluation by using a dose-response curve with linearity at a low dose range and a polynomial function with phase linearity for other doses. The results show that as the accumulated dose of the dosimeter increased, the responses were different from those in the low-dose range. However, if the dosimeters showed the same tendency, the dose-response curve, according to the dose range, would be useful. Furthermore, this study not only verified the possibility of evaluation for high doses, but also verified that the difference between the accumulated dose being irradiated repeatedly at the same dose without optical annealing, and the total dose being irradiated once, is within a valid range.

Furthermore, in a study on sensitivity changes for dosimeters irradiated with high doses, Han et al. found that as the dose was accumulated, the radiation sensitivity changed, and the sensitivity of the dosimeter, after irradiation with a high dose, became lower than its initial sensitivity. It was confirmed that the radiation sensitivity reduced linearly when the same dose was repeatedly irradiated in the OSLD. After irradiation with a high dose, the sensitivity of the OSLD reduced exponentially when the OSLD was repeatedly irradiated with the same dose. The user should recalibrate the OSLD’s sensitivity for reuse, considering its irradiation history [21]. Previous studies have shown that the sensitivity of the OSLD can be changed not only by doses, but also by the count value remaining after annealing, or by the annealing equipment and conditions [23, 24]. The measurements were also changed by the OSLD reading process and reading device. Thus, the American Association of Physicists in Medicine published the TG-191 report on this; it presented the measurement uncertainty that can occur when using dosimeters [16]. Considering these points, the present study designed a program for adding two factors: a factor for correcting errors that may occur in the reading system and a factor for correcting differences caused by the irradiation environment. We evaluated the irradiation environment with respect to most universal and basic environments. However, because the OSLD has a geometric dependence on the dosimeter [9], angular dependence can exist depending on the irradiation environment. The correction factors are summarized in the American Association of Physicists in Medicine (AAPM) TG-191. To minimize beam quality correction factors, it is sufficient to set the standard and evaluation irradiation conditions to be equal. Otherwise, the relative response of the OSLDs between different energies can be established A previous study evaluated the energy response of the OSLD normalized to their response at 6 MV(1 Gy), and the energy dependence was with 2% [15]. Hence, it is believed that the reliability of the measurement can be improved if this is considered. In addition, 60-Co gamma rays were used in this study. However, clinical radiation therapy uses beams of 6 MV or higher. Considering this, the accuracy of the measurement can be improved by adding a correction factor for energy.

In this study, the irradiation conditions were simplified as much as possible, to evaluate various dose ranges, including high doses. The dose evaluation using the OSLD was performed under the same conditions as obtaining the dose response curve. The exposure condition and reading system correction factor were not used. However, the above-mentioned factors were designed to be input into the developed program as user input. In the various radiation treatment environments of clinic radiotherapy, flexible dose evaluation will be possible with OSLDs and in-house programs. This study is a preliminary study for dose evaluation that considers various factors in various dose ranges using OSLDs. Additional studies are necessary on the changes in various irradiation environments for dose evaluation, such as irradiation field size, energy dependence, and incident angle. Based on the results of this study, we believe that various dose measurements using the OSLD will be possible through the use of an in-house software program and the aforesaid correction process.

## Supporting information

S1 File (ZIP)
