# Supplementary material for: Preliminary study for dose evaluation depending on dose range with optically stimulated luminescence dosimeter considering individual dosimeter sensitivity
Source: PLoS One. 2022 Mar 29;17(3):e0266110. doi: 10.1371/journal.pone.0266110 (PMC8963533; doi:10.1371/journal.pone.0266110)
Supplement: S1 File — (ZIP) [file pone.0266110.s001.zip › support_data1_Flowchart for development of in-house program.pptx]

## Slide 1
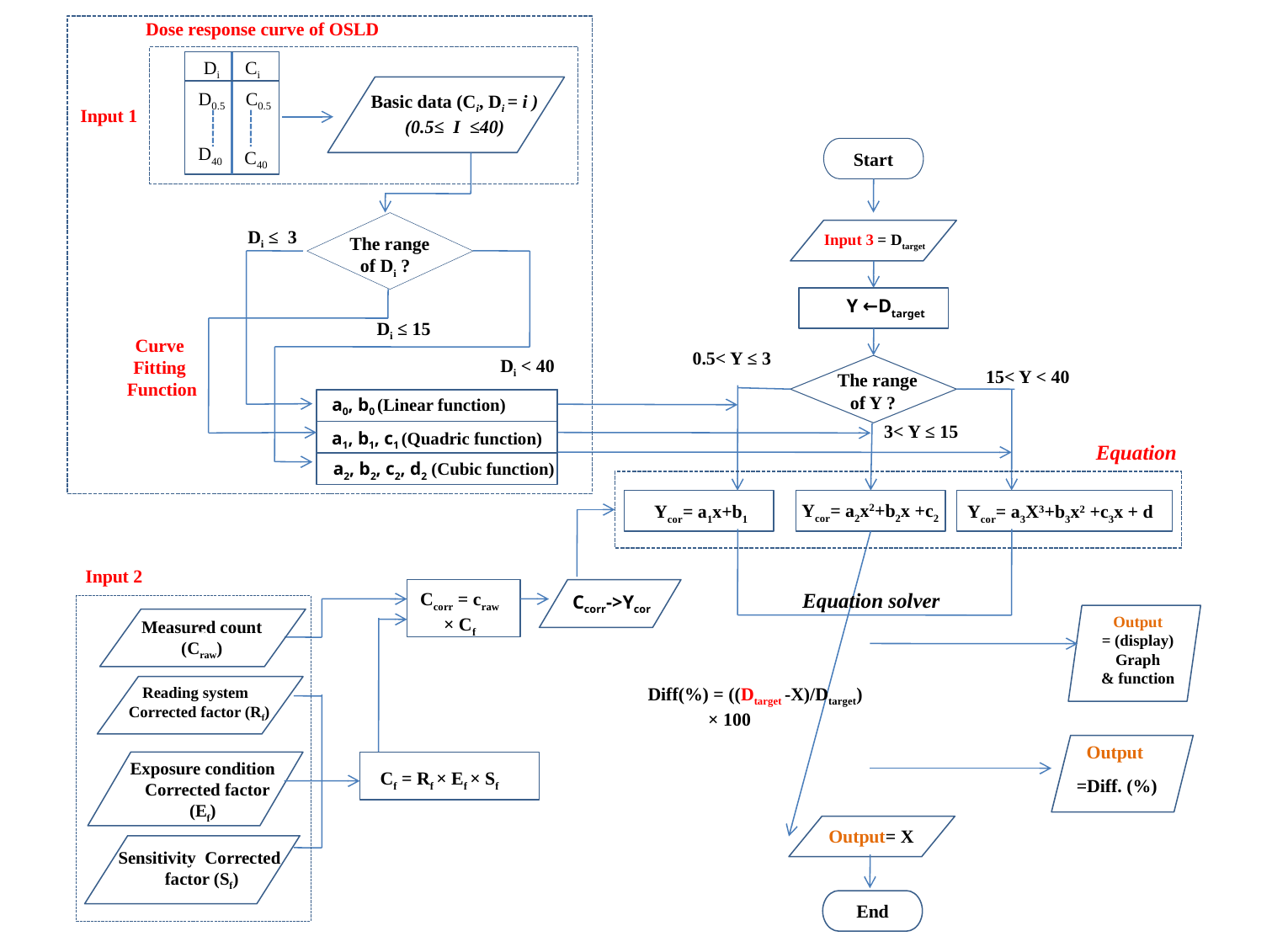

Dose response curve of OSLD
Di
Ci
D0.5
C0.5
D40
C40
I
Basic data (Ci, Di = i )
(0.5≤ I ≤40)
Input 1
Start
Di ≤ 3
I
Input 3 = Dtarget
The range
of Di ?
Y ←Dtarget
Di ≤ 15
Curve
Fitting
Function
0.5< Y ≤ 3
Di < 40
15< Y < 40
The range
of Y ?
a0, b0 (Linear function)
a1, b1, c1 (Quadric function)
a2, b2, c2, d2 (Cubic function)
3< Y ≤ 15
Equation
Ycor= a2x2+b2x +c2
Ycor= a1x+b1
Ycor= a3X3+b3x2 +c3x + d
Input 2
1
I
Equation solver
Ccorr = craw
× Cf
Ccorr->Ycor
Output
= (display)
Graph
& function
I
Measured count
(Craw)
Diff(%) = ((Dtarget -X)/Dtarget)
 × 100
Reading system
Corrected factor (Rf)
I
Output
Exposure condition
 Corrected factor (Ef)
I
Cf = Rf × Ef × Sf
=Diff. (%)
I
Output= X
Sensitivity Corrected
factor (Sf)
I
End
